# Clinically applied procedures for human ovarian tissue cryopreservation result in different levels of efficacy and efficiency

**DOI:** 10.1007/s10815-016-0816-z

**Published:** 2016-10-06

**Authors:** Lobke Bastings, Johan R. Westphal, Catharina C. M. Beerendonk, Ruud L. M. Bekkers, Petra L. M. Zusterzeel, Jan C. M. Hendriks, Didi D. M. Braat, Ronald Peek

**Affiliations:** 1 Department of Obstetrics and Gynecology, Radboud University Medical Center, PO Box 9101, 6500 HB Nijmegen, The Netherlands; 2 Department for Health Evidence, Section Biostatistics, Radboud University Medical Center, PO Box 9101, 6500 HB Nijmegen, The Netherlands

**Keywords:** Ovarian tissue, Cryopreservation, Fertility preservation, Efficiency, Efficacy

## Abstract

**Purpose:**

Different protocols are being used worldwide for the cryopreservation of human ovarian tissue for fertility preservation purposes. The efficiency and efficacy of the majority of these protocols has not been extensively evaluated, possibly resulting in sub-optimally cryopreserved ovarian tissue. To address the impact of this issue, we assessed the effects of two clinically successful human ovarian tissue slow-freezing cryopreservation procedures on the quality of the cryopreserved tissue.

**Methods:**

To differentiate between cryopreservation (_C_) versus thawing (_T_) related effects, four combinations of these two (A and B) very different cryopreservation/thawing protocols (A_C_A_T_, A_C_B_T_, B_C_A_T_, B_C_B_T_) were studied. Before and after cryopreservation and thawing, the percentage of living and morphologically normal follicles, as well as the overall tissue viability, was assessed.

**Results:**

Our experiments revealed that the choice of the cryopreservation protocol noticeably affected the overall tissue viability and percentage of living follicles, with a higher viability after protocol B_C_ when compared to A_C_. No statistically significant differences in tissue viability were observed between the two thawing protocols, but thawing protocol B_T_ required considerably more human effort and materials than thawing protocol A_T_. Tissue morphology was best retained using the B_C_A_T_ combination.

**Conclusion:**

Our results indicate that extensive and systematical evaluation of clinically used protocols is warranted.

## Introduction

The survival of pediatric, adolescent, and young adult cancer patients has significantly improved during the past decades [1]. As a consequence, the attention to issues related to quality of life after cancer has increased in oncological care. With many types of oncological therapy posing a threat to the ovarian function [2], techniques aimed at preserving fertility in girls and young women have emerged and evolved [3]. In this paper, we focus on the cryopreservation of ovarian tissue. In prepubertal girls, and in women who cannot delay the start of chemotherapy, this is the only available option for fertility preservation [4, 5]. The ovarian tissue is obtained before start of the gonadotoxic anti-cancer therapy and can be autotransplanted to the patient after she has been cured of her disease, to restore her fertility. In three major European centers for fertility preservation, a pregnancy rate of 27 % was reported after autotransplantation of ovarian tissue, and more than 60 babies have been born worldwide [5–7].

The freezing/thawing protocol used for cryopreservation of the ovarian tissue is most likely to be an important factor in determining the clinical outcome (i.e., live birth) of ovarian tissue autotransplantation. Several laboratory procedures that have led to the birth of healthy children have been published [8–17].

The mere fact that children were born using a certain protocol, however, does not automatically imply that this protocol had been optimized to its maximum potential. In other words, tweaking the protocol might have led to an even higher success rate. To address this matter, we compared the impact of two clinically successful, but methodologically very different freezing and thawing laboratory procedures on the quality of the cryopreserved tissue. To determine the contribution of the freezing and the thawing procedure on the human ovarian tissue quality, we analyzed the effects of these events separately.

## Materials and methods

### Study design and eligibility criteria

The effects of the two selected cryopreservation and thawing protocols: protocol A_C_A_T_ using ethylene glycol as cryoprotectant [18, 19] and B_C_B_T_ using DMSO as cryoprotectant [13, 20, 21] on overall ovarian tissue’s viability, follicle viability, and tissue morphology were investigated in a four-arm design:

- A_C_A_T_: cryopreservation and thawing protocol A
- B_C_B_T_: cryopreservation and thawing protocol B
- A_C_B_T_: cryopreservation protocol A followed by thawing protocol B
- B_C_A_T_: cryopreservation protocol B followed by thawing protocol A

For ethical reasons, we could not use tissue from young cancer patients applying for ovarian tissue cryopreservation. We therefore used ovarian tissue from premenopausal women aged ≤45 years who were considered eligible for this prospective cohort study if they underwent a prophylactic laparoscopic salpingo-oophorectomy at the Radboud university medical center (Radboudumc), Nijmegen, the Netherlands. Informed consent was obtained prior to surgery. When possible, cortex tissue from each patient was included in all four arms of the study. In addition, a fresh (positive) control was analyzed for each patient before cryopreservation/thawing. In case of insufficient material (due to size of the ovary, presence of large follicles, and/or extensive damage as a result of cauterization during surgery), the cortex tissue of a given patient was only used in three or less of the study arm protocols.

### Ethical approval

All study procedures were approved by the Radboudumc local ethics committee.

### Ovarian tissue preparation

At the operating theater, the ovary dissected during surgery was collected in cold Custodiol (4 °C; Dr. Franz Köhler Chemie GmbH, Bensheim, Germany). The ovary was immediately transferred to the laboratory (within 5–10 min), where it was placed on a pre-cooled surface (0 °C). The medulla was removed from the cortex using precision forceps and scalpels, after which ovarian cortex fragments of approximately 5 mm in length, 5 mm in width, and 1.0–1.5 mm in depth were prepared. These 5 × 5 × 1 mm fragments were subsequently used to prepare 3-mm-diameter punch biopsies for the glucose uptake assay (overall tissue viability) and for preparing small tissue fragments for the neutral red stain (follicle viability).

### Ovarian tissue cryopreservation and thawing

The ovarian tissue cryopreservation and thawing protocols selected for this study differ considerably. Briefly, protocol A consists of a cryopreservation protocol using ethylene glycol combined with a thawing protocol based on three consecutive short washes to remove the cryoprotectant. Protocol B uses dimethyl sulfoxide (DMSO) as a cryoprotectant and has a significantly longer and more elaborate thawing procedure based on continuous dilution. Tissue was stored in liquid nitrogen for at least 1 week before thawing.

As we combined two clinically used protocols in our study, we measured the osmolality of the four cryopreservation and thawing solutions in order to determine any additional osmotic stress to which the tissue was exposed when applying cryopreservation and thawing solutions from different protocols.

### Cryopreservation protocol A_C_

Tissue fragments were equilibrated in 30 mL of cryomedium, consisting of 0.1 mol/L sucrose (Sigma-Aldrich, Zwijndrecht, the Netherlands) and 1.5 mol/L ethylene glycol (Merck Millipore, Schiphol-Rijk, the Netherlands) in phosphate-buffered saline (PBS; Braun, Melsungen, Germany) for 30 min on a tilting table at 4 °C. Instead of using a Planer Freezer (Planer K10; Planer Ltd, UK), ovarian cortical fragments were frozen in Nunc CryoTubes (Sigma-Aldrich, St. Louis, MO, USA) in 1 mL cryomedium in a CryoLogic programmable temperature controller (CL-3300, Cryosolutions, ‘s Hertogenbosch, the Netherlands) using Cryogenesis^TM^ V5 software (Cryosolutions). CryoTubes were transferred to the CryoLogic set at 0 °C. Subsequently, the temperature was lowered at a rate of 2 °C/min until a temperature of −9 °C was reached. At this stage, CryoTubes were seeded manually with a cotton swab dipped in liquid nitrogen. The temperature remained at −9 °C for 10 min after which seeding was checked. Next, the temperature was decreased at a rate of 0.3 °C/min to −40 °C and subsequently with 8.5 °C/min to −120 °C before the CryoTubes was stored in liquid nitrogen. This freezing protocol was comparable to the original protocol, expect for the duration at which the temperature remained at −9 °C (5 versus 10 min) and the speed at which the temperature was lowered after reaching −40 °C (8.5 °C/min to −120 °C versus 10 °C/min to −140 °C in the original protocol; [18]). The cryomedium had an osmolality of 2.3 Osmol/kg.

### Thawing protocol A_T_

Tissue was thawed rapidly at 37 °C and then rinsed in 25 mL of three solutions (PBS containing 0.25 mol/L sucrose and 0.75 mol/L ethylene glycol; PBS containing 0.25 mol/L sucrose; and PBS, respectively) for 10 min each under continuous agitation to wash out the cryoprotectant. The first washing solution had an osmolality of 1.5 Osmol/kg. In total, this resulted in a thawing procedure of approximately 35 min.

### Cryopreservation protocol B_C_

According to cryopreservation protocol B_C_ [13, 20, 21], a freezing solution with an osmolality of 2.1 Osmol/kg was prepared consisting of Leibovitz’s L-15 GlutaMAX cryomedium (Gibco, Carisbad, CA, USA), containing 10 % dimethyl sulfoxide (1.4 mol/L; CryoSure-DMSO; Wak-Chemie Medical GmbH, Steinbach, Germany), and 10 % serum substitute supplement (SSS; Irvine Scientific, Santa Ana, CA, USA). Nunc CryoTubes were filled with 1.7 mL cold medium (0 °C) and a cortex fragment. Instead of using the program as described for the IceCube (14S-A, SY-LAB, Neupurkersdorf, Austria) in the original protocol [13], the CryoTubes were frozen in the CryoLogic programmable temperature controller using the program as stated under “Cryopreservation protocol A_C_.” This freezing protocol was comparable to the original protocol, expect for an incubation step in the programmable freezer at 2 °C for 40 min, the seeding temperature (−9 instead of −6 °C, allowing us to freeze tissue according to protocol A_C_ and B_C_ simultaneously and in the same Cryologic freezing device) and the speed at which the temperature was lowered after reaching −40 °C (8.5 °C/min to −120 °C versus 10 °C/min to −140 °C in the original protocol).

### Thawing protocol B_T_

Thawing protocol B_T_ was based on continuous dilution of the cryoprotectant [20]. The CryoTubes were removed from the liquid nitrogen, warmed for 30 s at room temperature, and placed in a 37 °C water bath for 2 min. Directly after thawing, the ovarian cortex fragments were transferred to a solution of 10 mL Dulbecco’s Phosphate-Buffered Saline (DPBS; Gibco), 0.75 M sucrose (MP Biomedicals, Eschwege, Germany), 10 % serum substitute supplement (SSS; Irvine Scientific, Santa Ana, USA), and 0.1 mg/mL Pen/Strep (Lonza, Basel, Switzerland) and incubated under continuous agitation for 15 min at room temperature. This first washing solution had an osmolality of 1.2 Osmol/kg. After this first incubation step, 50 mL of a solution consisting of DPBS with 10 % SSS and 0.1 mg/mL Pen/Strep was added using a pump set at 100 mL/h. At complete infusion (30 min), the cortex fragments were transferred into a 5 mL pre-warmed (37 °C) Hepes-buffered medium (Gamete, Cook Medical Europe LTD, Limerick, Ireland) and incubated for another 15 min. After repeating this step once, the tissue was washed three times for 5 min in tissue culture medium used for the glucose uptake assay (described below). In total, this resulted in a thawing procedure of approximately 1.5 h.

### Outcome measures

For the control as well as the four study arms, the overall ovarian tissue viability, follicle viability, and tissue morphology were examined using the following methods.

### Glucose uptake assay

The ovarian tissue’s overall viability was assessed using a glucose uptake assay we described previously [22]. This assay quantitatively measures the glucose uptake by ovarian tissue during in vitro culture. Glucose uptake has been shown to be inversely correlated with the extent of (cryo)damage the tissue has sustained [22]. This assay has also been successfully used for determining the viability of human ovarian cortex tissue after cryopreservation [21].

For each individual measurement, four cortex biopsies were prepared using a 3-mm-diameter biopsy punch (Pfm Medical ag, Cologne, Germany). For each of these four ovarian biopsies, glucose uptake was determined by culturing them separately in 2 mL Dulbecco’s Modified Eagle Medium High Glucose (4.5 g/L) with L-Glutamine (DMEM, PAA Laboratories) supplemented with 10% fetal bovine serum (FBS; PAA Laboratories GmbH, Cölbe, Germany) and 0.1 % Pen/Strep (GIBCO; 10.000 units penicillin/mL and 10.000 μg streptomycin/mL) in a 24-well plate (TPP, Trasadingen, Switzerland) at 37 °C in humidified air with 5 % CO_2_. The 2 mL of conditioned culture medium was collected from each individual biopsy, and replaced by fresh medium at day 4 and again collected at day 7, when the culture was ended. After culture, the biopsies were weighed separately and the glucose content of unconditioned medium (control) and of the conditioned medium after 4 or 7 days of culture were measured using an Architect i2000 system (Abbott Diagnostics, IL, USA). The glucose uptake of each biopsy during the culture period from day 0–4 and day 4–7 was determined by subtracting the glucose content of the conditioned medium from the unconditioned control medium. Glucose uptake was corrected for the weight (mg) of the biopsy and the duration (h) of the culture period [21, 22]. Then, for each series of four biopsies cultured under the same conditions, the mean glucose uptake/milligram/hour (nmol) was calculated representing an individual measurement on day 0–4, day 4–7, and (combined) day 0 to 7.

### Percentage of living follicles

**Fig. 1:**
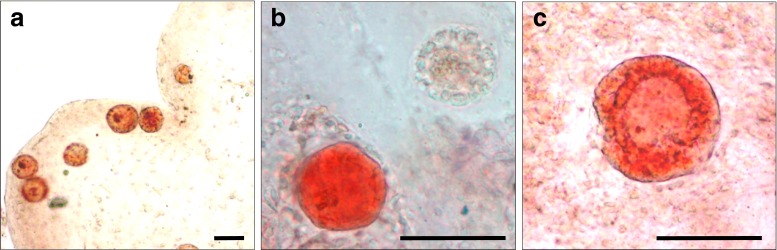
Follicles visualized by neutral red staining. **a** Overview of living follicles of various sizes as visualized with the neutral red viability stain. Due to the thickness of the squash preparation, not all follicles are in focus. **b** Fresh tissue with a viable follicle (*red*) and dead follicle (transparent). **c** Close-up of a living follicle. *Bars* represent 200 μM

The viability of ovarian follicles was assessed in fresh tissue, or immediately after thawing, using a neutral red (NR) assay modified from Kristensen et al. [23]. With this metabolic assay, based on the ability of living cells to incorporate and bind neutral red in their lysosomes [24], living follicles were stained red, whereas dead follicles remained transparent (Fig. 1). A total of 10–15 fragments (<1 mm^3^) were cut from 3–4 ovarian cortex fragments of 5 × 5 × 1 mm, and incubated in 5 mL Ultraculture (Lonza) supplemented with 1 mg/mL Collagenase type IA (Sigma-Aldrich, Steinheim, Germany) at 37 °C for 1 h to soften and partially dissolve the tissue. Subsequently, the suspension was centrifuged at 400×*g* for 5 min after which the tissue pellet was resuspended in NR solution, consisting of 4.8 mL McCoy medium (Gibco, Life Technologies, New York, USA), 75 μL neutral red (to a final concentration of 50 mg/mL; Sigma-Aldrich), 2 μL Albuman (200 g/L; Sanquin, Amsterdam, the Netherlands), 50 μL Insulin-Transferrin-Selenium (ITS-G, Gibco), 200 units penicillin (Gibco), and 200 μg streptomycin (Gibco). The NR suspension containing the ovarian tissue was incubated for 90 min at 37 °C in humidified air with 5 % CO_2_. Next, the partly dissolved tissue fragments were removed from the bottom of the tube and placed on a glass slide. By gently pressing a cover slip on the softened tissue fragments, a squash preparation was obtained. Living (stained red) as well as dead (non-stained) follicles were counted in this preparation using light microscopy. The percentage of living follicles was documented for each condition for which a NR viability assay was performed, provided that at least 20 follicles could be analyzed. Results obtained from samples with less than 20 follicles were considered unreliable and therefore excluded from further analysis. Using these criteria, the percentage of living follicles could be determined in the tissue of 16 patients while the tissue of the five remaining patients was excluded.

### Tissue morphology

For histology, cortex fragments of 5 × 5 × 1 mm from 18 patients were fixed in Bouin’s solution (Sigma-Aldrich) and subsequently embedded in paraffin wax. Eight μm hematoxylin- and eosin-stained sections were examined using light microscopy and photographed. For each fragment, three sections were evaluated. Sections were separated by 100 μm of tissue to prevent counting the same pre-antral follicle twice. Follicles were scored as morphologically normal or degenerated (in case of cytoplasmatic shrinkage, disorganized granulosa cells, pyknotic nuclei) and categorized as primordial/primary, secondary, or antral according to a predefined criteria [25]. We focused at the *percentage* of degenerated/dead follicles rather than the *total number* of follicles observed per study arm as the density of follicles in various parts of the cortex of the same ovary may vary considerably [26].

### Statistical methods

A linear-mixed model for repeated measurements was used to study differences between protocols used for cryopreservation and for thawing on each of variables of the viability of human ovarian tissue, separately. In this way, it is possible to find small within-subject differences relative to a large between-subject (biological) variation. The dependent variable was the glucose uptake and the percentage living follicles, respectively. The independent categorical variables were cryopreservation (two protocols A_C_, B_C_), thawing (two protocols A_T_, B_T_), and the time point of measurement (two levels day 0–4, day 4–7). The intercept of each patient was treated as a random variable, in order to allow different levels for different patients. Initially, all interaction terms between the categorical variables were included in the linear part of the model. However, these terms were omitted from the final model presented as these did not statistically significant improve the fit to the data (Likelihood-Ratio test). The estimated mean differences between the levels of each variable with the appropriate 95 % confidence interval are presented. Statistical analyses were performed by using SAS® version 9.2 for Windows (SAS institute Inc. Cary, NC, USA).

## Results

### Patient characteristics

A total of 21 women (median age 40 years; range 32–45) participated in this study. Twenty women were healthy BRCA 1 or 2 mutation carriers who did not receive prior gonadotoxic therapy, whereas one woman had her ovaries removed because of breast cancer after chemotherapy. Ten women used contraceptives (oral, *n* = 5; intrauterine device, *n* = 4; NuvaRing, *n* = 1), 10 had regular menstrual cycles, and one had had a hysterectomy in the past. This last participant was considered premenopausal as she had a normal level of anti-Mϋllerian hormone (AMH 3.2 μg/L) without menopausal symptoms. For four women, only part of the study arms could be evaluated, because of a small ovary (*n* = 2), the presence of large follicles (*n* = 1), or cauterization damage (*n* = 1).

### Glucose uptake

In Table 1, the observed median glucose uptake per milligram ovarian tissue per hour of culture is presented for fresh ovarian tissue, and for ovarian tissue subjected to one of the four different combinations of freezing (A_C_ and B_C_) and thawing protocols (A_T_ and B_T_), expressed as mean of 4 separately assayed cortex fragments per individual patient (*n* = 18–21; see Table 1). Biopsies showed no difference in weight between cultured biopsies and fresh biopsies (data not shown).

**Table 1:** The observed median (range) of the glucose uptake and the percentage of living follicles for each combination of cryopreservation protocol and thawing protocol

Protocol		Glucose uptake (nmol/mg/h)					Living follicles (%)		
Cryopreservation	Thawing	*N*	Culture days 0–4 median (range)		Culture days 4–7 median (range)		*N*	median (range)	
Fresh	Fresh	21	23.5	(8.3–32.7)	20.3	(8.8–28.3)	11	94.5	(89.1–98.3)
A_C_	A_T_	18	8.7	(−0.5–29.5)	8.1	(1.0–26.8)	16	72.5	(26.3–95.4)
A_C_	B_T_	19	7.1	(−0.5–25.3)	6.5	(−5.5–29.0)	15	76.6	(20.0–95.7)
B_C_	A_T_	20	17.3	(0.8–31.3)	18.2	(0.5–30.9)	17	87.5	(65.5–96.1)
B_C_	B_T_	20	16.3	(1.9–24.1)	16.8	(3.2–23.9)	14	88.7	(18.2–100.0)

The glucose uptake is expressed in nanomolars per milligram of ovarian cortex tissue per hour of culture. Follicle viability assessment by the neutral red staining was performed in fresh tissue or immediately after thawing. The percentage of living follicles was only determined in preparations in which at least 20 follicles could be counted

*A*_*C*_ cryopreservation protocol A, *A*_*T*_ thawing protocol A, *B*_*C*_ cryopreservation protocol B, *B*_*T*_ thawing protocol B, *N* number of patients

Glucose uptake was comparable for the day 0–4 culture and the 4–7 day culture period and was not significantly different (Tables 1 and 2). For reasons of clarity, only the day 0–4 results are described.

**Table 2:** The estimated mean difference (95 % confidence interval (CI)) of the glucose uptake and the percentage of living follicles between the cryopreservation protocols, thawing protocols, and culture periods, using a linear-mixed model for repeated measurements

	Protocol/day	Glucose uptake (nmol/mg/h)			Living follicles (%)		
		Estimated difference			Estimated difference		
		mean (95 % CI)		*p* value	mean (95 % CI)		*p* value
Cryopreservation	A_C_	0.0	Reference		0.0	Reference	
	B_C_	6.8	(5.0; 8.6)	<0.001	11.3	(4.6; 18.0)	0.002
Thawing	A_T_	0.0	Reference		0.0	Reference	
	B_T_	−1.7	(−3.5; 0.0)	0.051	−1.5	(−8.1; 5.1)	0.651
Culture period	Day 0–4	0.2	(−1.6; 1.9)	0.842			
	Day 4–7	0.0	Reference				

*A*_*C*_ cryopreservation protocol A, *A*_*T*_ thawing protocol A, *B*_*C*_ cryopreservation protocol B, *B*_*T*_ thawing protocol B

As expected, the highest glucose uptake was found for fresh tissue (23.5 nmol/mg/h). Note that the range of glucose uptake by the tissues derived from individual patients was large (range 8.3–32.7 nmol/mg/h), demonstrating a large inter-patient variability. Independent of the thawing protocol, tissue cryopreserved according to protocol B_C_ had a significantly higher glucose uptake when compared to A_C_ (B_C_B_T_ 16.3 and B_C_A_T_ 17.3 vs A_C_B_T_ 7.1 and A_C_A_T_ 8.7 nmol/mg/h, Table 1). In Table 2, the estimated differences of the glucose uptake between the cryopreservation and thawing protocols—as obtained by a linear-mixed model for repeated measurements—are presented. According to this model, tissue that was cryopreserved using protocol B_C_ showed a statistically significant higher glucose uptake (6.8 nmol/mg/h higher; *p* < 0.001) than tissue cryopreserved using protocol A_C_ (Table 2). A small difference in glucose uptake (1.7 nmol/mg/h) was found in favor of the shorter thawing protocol A _T_ when compared to B_T_, but this difference did not reach statistical significance (*p* = 0.051; Table 2).

**Fig. 2:**
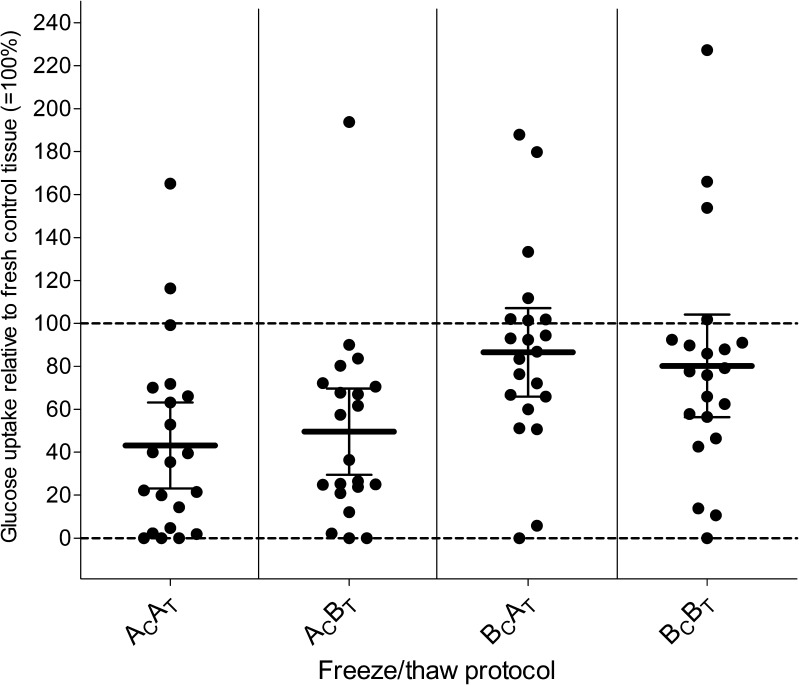
Glucose uptake by ovarian cortex tissue after different combinations of cryopreservation and thawing protocols. Total glucose uptake during 7 days of culture is expressed as a percentage of glucose uptake by fresh (=non-frozen, non-thawed) ovarian cortex tissue of the same patient. Mean and 95 % confidence interval are indicated. *A*_*C*_ cryopreservation protocol A, *A*_*T*_ thawing protocol A, *B*_*C*_ cryopreservation protocol B, *B*_*T*_ thawing protocol B

To eliminate the large inter-patient variability in glucose uptake mentioned previously, we also expressed glucose uptake of frozen and thawed ovarian tissue as a percentage of the glucose uptake by fresh control tissue (Fig. 2). As already observed for the absolute values of glucose uptake levels in Table 1, the decrease in glucose uptake was considerably less in tissue frozen via the B_C_ protocol (19.8 and 13.5 % decrease for the B_C_B_T_ and B_C_A_T_ protocol, respectively), compared to tissue frozen via the A_C_ protocol (50.4 and 56.8 % decrease for the A_C_B_T_ and A_C_A_T_ protocol, respectively). The decrease in glucose uptake was independent of the thawing protocol. Remarkably, we observed a post freeze/thaw increase in glucose uptake in comparison to the glucose uptake by the corresponding fresh control tissue in small subset of patients (1–5 patients per freeze/thaw protocol combination).

### Percentage of living follicles

**Fig. 3:**
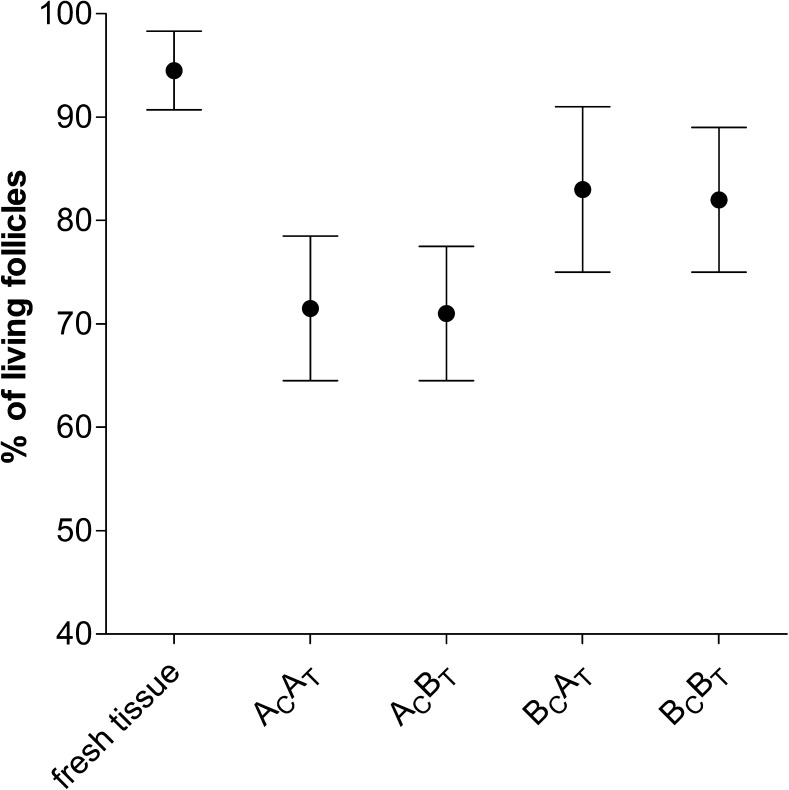
Percentage of living follicles in ovarian tissue after different combinations of cryopreservation- and thawing protocols. Follicle viability was assessed by neutral red staining. Results are expressed as percentage living follicles of the total number of follicles that was counted, and expressed as mean and 95 % confidence interval

A mean number of 80 follicles (SD 49) was evaluated in each ovarian tissue sample using the neutral red viability assay after having excluded samples with less than 20 follicles (*n* = 21 out of *n* = 98). Similar numbers of follicles could be counted before and after excluding the samples with less than 20 follicles for each of the study arms (data not shown). In Table 1, the observed percentages of living follicles obtained with the neutral red viability assay are presented. In accordance with the results regarding the tissue’s glucose uptake, tissue that was cryopreserved according to protocol B_C_ showed a statistically significant higher percentage of living follicles when compared to tissue cryopreserved according to protocol A_C_ (p=0.002; Table 2 and Fig. 3). Analogously to the results of the glucose uptake assay, we observed no significant difference between thawing protocols A_T_ and B_T_ with respect to the percentage of living follicles (p=0.651; Table 2).

We found the correlation between glucose uptake and follicle viability to be poor in all four study arms during 0–4 and 4–7 day culture (Spearman’s correlation <0.3, *p* values 0.33–0.99).

### Follicle morphology

**Fig. 4:**
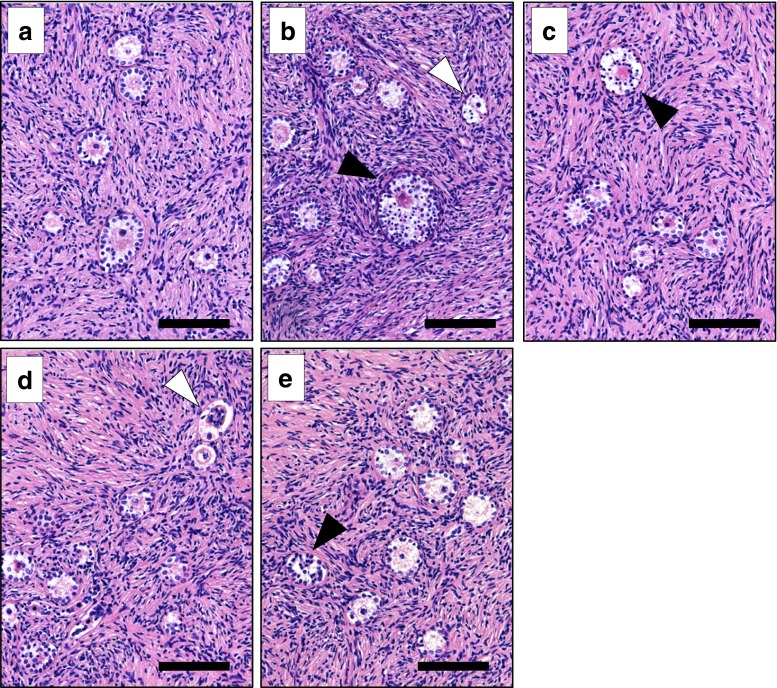
Follicle morphology after different combinations of cryopreservation and thawing protocols. Follicle morphology was determined by analysis of hematoxylin-/eosin-stained sections of ovarian cortex tissue before (panel **a**) and after cryopreservation and thawing according to cryopreservation protocol A_c_A_T_ (panel **b**), protocol A_c_B_T_ (panel **c**), protocol B_c_B_T_ (panel **d**), and protocol B_c_A_T_ (panel **e**). In panels **b**–**e**, the follicles with pyknotic nuclei (*black arrow heads*) and cytoplasmic shrinkage (*white arrow heads*) are indicated. *Bars* represent 100 μM

Histological examination of the hematoxylin- and eosin-stained sections from fresh tissue revealed that 99 % of the follicles were morphologically normal. After cryopreservation/thawing according to the four study arms, 89 % (A_C_A_T_), 85 % (A_C_B_T_), 93 % (B_C_A_T_), and 84 % (B_C_B_T_) of the primordial/primary follicles were morphologically normal, whereas the remaining primordial/primary follicles showed cytoplasm shrinkage, disorganized granulosa cells, or pyknotic nuclei as a sign of follicle degeneration (Fig. 4). The majority of the total of 2039 follicles observed were in the primordial or primary stages. For each of the four study arms as well as the fresh tissue, 2–3 % of the follicles were in their secondary or antral stages. The percentage of degenerated secondary follicles was similar to the percentage degenerated primordial/primary follicles. Most of the follicles (>90 %) in the antral stage showed some signs of degeneration after freezing and thawing.

## Discussion

Currently, many different protocols are used worldwide for the cryopreservation and subsequent thawing of human ovarian tissue intended for fertility preservation. Little is known regarding the relative efficiency and efficacy of these protocols. To investigate this matter, we compared two human ovarian tissue slow-freezing cryopreservation and thawing protocols that have both proven to be clinically successful. Our explicit goal was to explore any possible differences in efficiency and efficacy between protocols that are clinically used, rather than to identify a superior freezing/thawing protocol.

The glucose uptake by ovarian tissue derived from different patients showed a large degree of variability (Table 1). This was also observed in a previous study [21]. These differences may be explained by ovarian cortex tissue heterogeneity between different patients or, alternatively, reflect differences in the initial viability of the tissues due to differing periods of warm ischemia after the surgical removal of the ovary.

We observed significant differences between two protocols for freezing human ovarian tissue with regard to their effect on glucose uptake. We have previously shown that glucose uptake is strongly correlated with tissue viability [21, 22]. A better tissue viability, as observed after applying protocol B_C_ for cryopreservation of human ovarian tissue, may translate in improved efficacy, i.e., an increase in the number of live births.

Several differences between cryopreservation protocols A_C_ and B_C_ can be identified; the major one being the use of ethylene glycol as a cryoprotectant in protocol A_C_ and DMSO in protocol B_C_.

Previous studies have compared DMSO and ethylene glycol—in the absence or presence of sucrose—in slow-freezing protocols for the cryopreservation of animal or human ovarian tissue or isolated follicles. In accordance with the results of the current study, a better follicle morphology and a higher percentage of living follicles were found after cryopreservation using DMSO when compared to ethylene glycol for human, sheep, or goat ovarian tissue [27–30]. However, DMSO did not improve the preservation of the follicle survival and ultrastructure when compared to ethylene glycol in bovine or agouti tissue [31, 32].

The two thawing procedures we used were very different with respect to the composition of the thawing solutions, as well as the length of the thawing process. This makes it difficult to separately evaluate the effects of the factor time and of the various components used in the thawing solution and, consequently, to pinpoint the individual contribution of these two factors on the efficacy of the thawing procedure. The exact reason for the effects on thawing efficacy notwithstanding, it is clear that protocol A_T_ was more time effective and required less human effort and material than protocol B_T_ and can therefore be considered more efficient.

Interestingly, the percentage of morphologically normal follicles was generally higher than the percentage of viable follicles. This may due to the fact that follicles that appear morphologically normal are, due to the freezing and thawing procedure, not metabolically active anymore.

We found no correlation between follicle viability and levels of glucose uptake. This is not surprising as stromal cells contribute by far the most to the cellular volume of the ovarian cortex. As a consequence result from the glucose uptake, assay will predominantly reflect the viability of the stromal compartment. We have shown that stromal cells are more sensitive to the damaging effects of the freeze thaw/procedure than follicles (as demonstrated by the decrease in glucose uptake after freeze/thawing, and the decrease in viable follicles, respectively). Similar findings were reported by Kim et al. [33] and Sanfilippo et al. [16]. Stromal cells are essential for the neovascularization of an ovarian graft after autotransplantation [34] and thus for follicle growth and maturation after transplantation. In this context, including viability of the stromal compartment in the evaluation of cryopreservation and thawing protocols, it is essential.

Several factors may have influenced the outcome of our study. First, the donors of ovarian tissue (BRCA mutation carriers) are considerably older than the population eligible for cryopreservation of ovarian tissue. Several authors have shown that the composition and structure of ovarian tissue alters as a result of aging [35, 36]. Consequently, the effects of various cryopreservation and thawing protocols should be further evaluated in a population of young patients applying for fertility preservation. Secondly, the percentage of living follicles observed after NR stain may be overestimated as the dead, non-stained follicles are less visible and might be missed during analysis. Finally, our experiments included combining a freezing solution containing DMSO and a thawing solution with ethylene glycol. Obviously, this will never be the combination of choice, as this will result in different rates of efflux and influx of two different cryoprotectants into the cells. However, we decided to test these combinations to study the effect of freezing and thawing separately.

Cryopreservation and thawing protocols might be modified or optimized over time. For example, human ovarian tissue was initially cryopreserved without a protein source in the cryopreservation medium in protocol A_C_A_T_ [18]. In a more recent overview presented by the same group, however, the otherwise identical cryopreservation medium was described to contain 1 % serum albumin [19]. The impact of this change in protocol was not evaluated by the team using the protocol in clinical practice and it is therefore unknown to what extent this modification would have changed the results of our current study. Obviously, it remains unknown from our data to what extent the impairment of the ovarian tissue viability observed in this study influences pregnancy rates after autotransplantation.

In conclusion, we demonstrate considerable differences in the effects of two clinically successful freezing and thawing protocols on the viability of human ovarian tissue, in a series of 21 patients. More precisely, the efficacy of the freezing protocols and the efficiency of the thawing procedures proved to be very different. To the best of our knowledge, this is the first report on human ovarian tissue cryopreservation that provides information on the effectiveness of the cryopreservation and the thawing procedures separately. Once again, we would like to emphasize that it was not our intention to discover the ultimate cryopreservation protocol. Rather, we want to stress that there actually are, with regard to efficacy and efficiency, considerable differences between clinically successful cryopreservation protocols. As a consequence, further optimization of these protocols may lead to even higher success rates.
